# Talking matters – evaluative and motivational inner speech use predicts performance in conflict tasks

**DOI:** 10.1038/s41598-019-45836-2

**Published:** 2019-07-02

**Authors:** Miriam Gade, Marko Paelecke

**Affiliations:** 1 Catholic University of Eichstätt-Ingolstadt, Department of Psychology, Eichstätt, D-85072 Germany; 2 Julius Maximilians University of Würzburg, Department of Psychology I, Würzburg, D-97070 Germany; 3 Medical School Berlin, Department of Sciences, Berlin, D-12247 Germany

**Keywords:** Human behaviour, Working memory, Cognitive control, Personality, Attention

## Abstract

Conflict between response tendencies is ubiquitous in every day performance. Capabilities that resolve such conflicts are therefore mandatory for successful goal achievement. The present study investigates the potential of evaluative and motivational inner speech to help conflict resolution. In our study we assessed six tasks commonly used to measure conflict resolution capabilities and cognitive flexibility in 163 participants. Participants additionally answered questionnaires concerned with their habitual usage of inner speech such as silently rehearsing task instructions and evaluating performance. We found reduced conflict effects in tasks using symbolic, non-verbal stimuli for participants with higher self-reported use of evaluative and motivational inner speech. Overall, our findings suggest that silent self-talk and performance monitoring are beneficial for conflict resolution over and above constructs such as intelligence and working memory capacity that account for mean RT differences among participants.

## Introduction

In everyday tasks, we often encounter opposing response tendencies, which lead to the experience of conflict^1–3^. Mechanisms for conflict resolution and in consequence goal achievement are commonly subsumed under the umbrella term of cognitive control. Investigating the processes underlying cognitive control, frameworks incorporating processes such as inhibition of irrelevant information or switching of attention have been proposed (e.g., by Friedman and Miyake^4^). In another line of (developmental) research, Luria^5^ highlighted the role of language for successful cognitive control and goal achievement. Likewise, Vygotskij^6^ argued that language and especially the extended representational power it represents, accelerates cognitive development and allows for more effortful processing, for instance inhibiting irrelevant information. Recently, Gruber and Goschke^7^ emphasized the close link between language and cognitive control, assuming verbal representations underlying performance in tasks that require conflict resolution (i.e., conflict tasks). Yet so far, surprisingly little research has been done to investigate the impact of individual preferences for language use and its benefits for conflict tasks in healthy adult participants. Mostly this research has focused on paradigms in which participants were either instructed to use language to help performance^8^ or asked to perform a secondary articulation task. In these studies it was found that language either helped or interfered with the primary task requirements^9–12^. Habitual usage of inner speech as a personality trait has not been considered as a predictor for performance in conflict tasks, different to other fields such as athletic performance^13^.

Inner speech, i.e., the subjective experience of self-directed language without audible articulation^14^ is usually investigated in contexts of health psychology, related to self-regulation^15^ and psycho-social well-being^16–18^. Starting from the ideas of Luria and Vygotski, we predicted that habitually talking to oneself during conflict tasks is beneficial for conflict resolution. Recently, we investigated habitual inner speech in a Simon task. We found that habits related to motivational (i.e., “you can do that”) and evaluative (i.e, “well done”) inner speech predict the size of the Simon effect^19^. The Simon effect reflects the difference in performance for trials in which the task-irrelevant position of the stimulus on the screen mismatches with the to-be given response compared to trials in which stimulus-location and response-location match. Those participants who engage more in motivational and evaluative inner speech had smaller Simon effects compared to less engaged participants, suggesting better inhibition of irrelevant information. These results lend support to the hypothesis that task-related contents of inner speech, i.e. instructing oneself or evaluating task performance, help to maintain focus on task-relevant features and resolve response conflict. We assume that both, motivational aspects (i.e., instructing oneself silently which key to press) as well as evaluative aspects (i.e., monitoring accuracy of behaviour and commenting on it), are relevant for performance benefits and predict smaller conflict effects for participants relying on inner speech in paradigms commonly used to investigate conflict resolution capabilities.

For the present study, we opted for tasks that rely on endogenous regulation of control. In addition, we assessed two switching tasks. We chose this addition to investigate a wider range of presumed cognitive control processes^4^ that might be susceptible to inner speech habits. We reasoned that inner speech might not only help to resolve conflict but also foster cognitive flexibility assessed in the switching tasks. Next to the overall requirement of conflict resolution, the specific task characteristics (i.e., stimulus material) may modulate the usage of inner speech. Therefore, we assessed tasks using verbal stimulus material such as words and tasks that employed figural/symbolic stimulus material such as shapes or arrows only. Our predictions were that modulation of behavior due to inner speech processes should be less detectable in tasks using verbal material as most participants would engage in covert articulation to guide performance (i.e., rehearsing stimulus-response mappings). In contrast, in non-verbal conflict tasks, retrieving nonverbal symbolic representations does not necessarily require inner speech, making global or task-related motivational and evaluative inner speech helpful to maintain focus and effort in the ongoing task. Therefore, individual differences in influences of inner speech should be readily visible in those tasks.

The nonverbal tasks chosen comprised the Simon task^20^ that affords a reaction towards a stimulus feature such as shape while ignoring the stimulus position and the flanker task^21^ for arrows in which participants had to react to the direction of the central arrow and ignore the flanking arrows. The verbal tasks chosen comprised the Stroop task^22^ that required the identification of the ink color in which a color word was printed while ignoring its meaning and the flanker task^21^ for letters where participants had to react to the identity of the central letter and ignore the flanking letters. The task-switching tasks comprised a non-verbal and verbal version in which either shapes or words had to be classified.

In all conflict tasks the amount of conflict is assessed as the difference between congruent (i.e., flankers and targets match) and incongruent (flankers and targets mismatch) trials. The main dependent variable in the switching task is the switch cost, i.e., the difference between task repetition and task switch trials. We predicted reduced conflict effects and switch costs in participants relying more on inner speech. Those reductions should also be more obvious in tasks not employing verbal material. We also reasoned that not all types of inner speech behavior might be equivalently helpful (see for instance, detrimental effects of rumination behavior^17^). Therefore, motivational and evaluative inner speech should have the biggest influence as these are closely linked to required behavior (see also^19^).

Inner Speech was assessed in quantity and quality using three questionnaires (Varieties of Inner Speech Questionnaire [VISQ]^15^; Self Talk Scale [STS]^16^, and the Inner Speech Scale [ISS]^23^). Additionally, we measured general intelligence (IQ) and working memory capacity (WMC) as control variables as the size of conflict effects are bound to overall mean RTs^24,25^. Controlling for variables known to affect mean RT such as intelligence and working memory capacity therefore seemed prudent. To sum up, starting from ideas of close linkage between language use and cognitive control we hypothesized that participants habitually engaging in inner speech processes should show reduced conflict effects and smaller switch costs. This reduction should be especially evident in task using non-verbal stimulus material as the involvement of verbal recoding enhances task-underlying representations and render them susceptible for proactive control processes that reduce the impact of conflicting response tendencies^26^. This predictive value of inner speech habits should exist incrementally over and above differences in intelligence and working memory capacity.

## Results

### Factor structure of the inner speech questionnaires and correlations with big five, depression, working memory capacity and intelligence

We originally had planned a hierarchical measurement model with one latent variable accounting for the shared variance of all inner speech scales (see registration, https://osf.io/xeth7/), based on the idea of a higher-order component of inner speech frequency^16^. VISQ and STS subscales, however, were only weakly correlated (mean $r=0.17$), leading to an unacceptable fit of the preregistered one-factor measurement model (${\rm{CFI}}=0.80$, ${\rm{RMSEA}}=0.14$). Separate confirmatory factor analyses (CFAs) for the VISQ and STS showed acceptable fit for models with latent factors for each of the four assumed subscales and the corresponding items as manifest variables (VISQ, ${\rm{CFI}}=0.92$, ${\rm{RMSEA}}=0.058$; STS ${\rm{CFI}}=0.93$, ${\rm{RMSEA}}=0.065$), replicating the factor structures of the original versions. Single factor models did not yield acceptable fit (VISQ, ${\rm{CFI}}=0.46$, ${\rm{RMSEA}}=0.149$; ${\rm{STS}}\,{\rm{CFI}}=0.80$, ${\rm{RMSEA}}=0.136$; ISS ${\rm{CFI}}=0.85$, ${\rm{RMSEA}}=0.093$), precluding the use of total scores especially for the VISQ but also for the STS and the ISS. For the subsequent analyses we therefore used the subscale scores of the VISQ and STS.

There were no meaningful correlations of the inner speech scales with the Big 5 (mean $r=0.02$ for the VISQ subscales and $r=-\,0.01$ for the STS subscales). There were small positive correlations of the inner speech scales with the ADS (mean $r=0.14$ for the VISQ subscales and $r=0.16$ for the STS subscales). In the sample of participants that took part in the lab study, none of the inner speech subscales correlated with WMC (mean $r=0.01$ for the VISQ subscales and mean $r=0.00$ for the STS subscales) or IQ (mean $r=0.09$ for the VISQ subscales and mean $r=-\,0.06$ for the STS subscales). For full correlation matrizes between predictors and control variables see Tables S1–S2 in Supplementary materials.

### Predicting task performance

The variances and covariances of the response times (RTs) in the four conflict and two task switching tasks were analyzed by Hierarchical Linear Modeling^27^ using HLM7^28^. Level 1 modeled the within-subjects variability by predicting each single RT from a dummy variable for each task coding general RT and a task-specific contrast that modeled the RT difference between congruent and incongruent trials with in each task. The congruency contrasts were coded +0.5 for incongruent trials and −0.5 for congruent trials, implying that a positive coefficient indicates slower responses in incongruent compared to congruent trials (i.e., a Simon effect). Level 2 modeled between-subject variability of the task-specific parameters coding general RT and conflict. Split-half reliability of the individual participants’ task parameters and their correlations are depicted in Table 1.

**Table 1:** Task parameter reliabilities (diagonal) and their correlations (above the diagonal).

Task parameter	1	2	3	4	5	6	7	8	9	10	11	12
general RT												
1. Simon	0.94	0.71	0.76	0.67	0.79	0.66	−0.33	0.38	0.26	0.69	−0.08	0.17
2. Arrow flanker		0.90	0.57	0.61	0.59	0.59	−0.10	0.79	0.22	0.58	0.24	0.14
3. Pictorial switching			0.94	0.56	0.62	0.70	−0.50	0.24	0.38	0.61	0.12	0.20
4. Stroop				0.93	0.65	0.58	0.12	0.37	0.11	0.77	0.03	0.13
5. Letter flanker					0.93	0.67	−0.23	0.40	0.09	0.63	0.12	0.03
6. Verbal switching						0.91	−0.48	0.39	0.19	0.49	0.41	0.35
conflict												
7. Simon							0.87	0.14	−0.28	0.10	−0.21	0.17
8. Arrow flanker								0.74	0.03	0.53	0.18	0.03
9. Pictorial switching									0.90	0.05	−0.25	0.28
10. Stroop										0.73	−0.04	0.14
11. Letter flanker											−0.09	0.16
12. Verbal switching												0.64

*Note*. *N* = 155. Critical values of |*r*| for *p* < 0.05 are *r*_*c*_ = 0.13 (one-tailed) and *r*_*c*_ = 0.16 (two-tailed).

We expected higher correlations of the conflict parameters within the nonverbal vs. verbal tasks than across these tasks, suggesting different amounts of reliance on inner speech. Whereas intercorrelations of the task parameters were high for general RT (mean $r=0.65$), they were very low for conflict (mean $r=-\,0.04$ for nonverbal tasks, and $r=0.09$ for verbal tasks). The preregistered measurement model with two latent factors accounting for the shared variance of conflict in nonverbal tasks vs. verbal tasks and empirical Bayes estimates of the six task-specific conflict parameters as manifest variables had an unacceptable fit (${\rm{CFI}}=0.74$, ${\rm{RMSEA}}=0.24$). Fit was only marginally better for a one-factor model (${\rm{CFI}}=0.75$, ${\rm{RMSEA}}=0.22$) and a model with two latent factors accounting for the shared variance of conflict in inhibition (Simon, Stroop, flanker) vs. switching tasks (${\rm{CFI}}=0.79$, ${\rm{RMSEA}}=0.21$). These results led us to abandon the pre-registered measurement model and adopt a piecewise analysis strategy in line with recent research^29–31^ that observed similarly low intercorrelations yet good reliabilities.

For the piecewise analyses we included the particiants WMC, IQ as well as inner speech questionnaire scores in the level-2 model and simultaneously predicted the individual participants’ task-specific parameters for general RT and conflict for all six task. We ran two separate analyses for the subscale scores of the VISQ and STS. The coefficients for fixed effects of general RT and conflict for each task are depicted in Tables 2 and 3 (for error analysis see Tables S5–S6 in Supplementary material). Scores of all level-2 predictors were entered as standard scores ($M=0$, $SD=1$). Thus, the level-2 coefficients indicated the time in milliseconds participant’s general RT (i.e., the dummy variable for each task) or the RT increment for incongruent compared to congruent trials (i.e., the task-specific conflict parameter) changed if the WMC, IQ or questionnaire score increased by one standard deviation.

**Table 2:** Coefficients (robust standard errors) for fixed effects of general RT and conflict for each task, with participants WMC, IQ as well as their VISQ subscale scores as simultaneous predictors in the Level-2 model.

Task		Intercept		WMC		IQ		VISQ							
								DLG		CND		OTP		E/M	
nonverbal															
Simon	general RT	**544**	(7)	−11	(8)	−**28**	(10)	−1	(8)	13	(7)	7	(9)	4	(9)
	conflict	**6**	(3)	−1	(5)	0	(3)	2	(2)	−2	(3)	4	(3)	−**6**	(3)
flanker	general RT	**515**	(6)	−1	(8)	−**30**	(10)	5	(6)	**20**	(8)	12	(10)	−13	(8)
	conflict	**98**	(4)	−7	(6)	−**11**	(4)	4	(4)	9	(6)	6	(4)	−**10**	(5)
switching	general RT	**958**	(19)	−9	(24)	−**62**	(25)	6	(21)	**40**	(18)	10	(20)	13	(21)
	conflict	**109**	(7)	12	(8)	−11	(9)	8	(7)	11	(7)	4	(8)	2	(7)
verbal															
Stroop	general RT	**648**	(11)	−5	(14)	−**51**	(17)	−3	(10)	13	(11)	16	(14)	−3	(13)
	conflict	**39**	(3)	−8	(4)	−6	(4)	−2	(3)	3	(4)	4	(3)	−1	(4)
flanker	general RT	**622**	(9)	−22	(12)	−20	(11)	3	(9)	3	(10)	5	(10)	−1	(10)
	conflict	**26**	(2)	3	(3)	−3	(3)	−2	(2)	−1	(2)	3	(2)	0	(2)
switching	general RT	**1031**	(18)	−36	(23)	−**72**	(22)	13	(20)	33	(18)	23	(21)	13	(23)
	conflict	**130**	(7)	**21**	(9)	−**25**	(9)	13	(7)	14	(8)	**17**	(8)	1	(8)

*Note*. *N* = 144, approx. *d*.*f*. = 137. WMC Working Memory Capacity, IQ General Intelligence, VISQ: DLG = dialogic inner speech, i.e. talking to oneself going back and forth, CND = condensed inner speech, i.e., shortcutting sentences, using brief phrases, OTP = other people in inner speech, i.e., hearing other people talk in my head, E/M = evaluative and motivational inner speech, i.e. telling myself not to do things. Significant coefficients (*p* < 0.05) are printed bold.

**Table 3:** Coefficients (robust standard errors) for fixed effects of general RT and conflict for each task, with participants WMC, IQ as well as their STS subscale scores as simultaneous predictors in the Level-2 model.

Task		Intercept		WMC		IQ		STS							
								SAS		SRE		SCR		SMA	
nonverbal															
Simon	general RT	**544**	(7)	−15	(8)	−**26**	(10)	−9	(10)	5	(10)	17	(11)	2	(11)
	conflict	**6**	(3)	−1	(5)	0	(3)	3	(3)	**7**	(3)	−**7**	(3)	−3	(4)
flanker	general RT	**515**	(6)	−2	(8)	−**30**	(11)	17	(9)	−6	(11)	−2	(9)	−13	(10)
	conflict	**98**	(4)	−7	(6)	−**12**	(5)	**14**	(7)	−1	(7)	0	(6)	−**18**	(9)
switching	general RT	**958**	(20)	−15	(26)	−**54**	(26)	−10	(26)	17	(26)	21	(26)	−3	(29)
	conflict	**109**	(7)	10	(8)	−9	(10)	−12	(10)	4	(9)	8	(10)	0	(12)
verbal															
Stroop	general RT	**648**	(11)	−10	(15)	−**50**	(18)	−19	(16)	19	(14)	1	(17)	1	(16)
	conflict	**39**	(3)	−8	(4)	−6	(3)	−1	(4)	−4	(4)	−1	(4)	0	(5)
flanker	general RT	**622**	(9)	−**24**	(12)	−16	(11)	1	(12)	9	(11)	3	(13)	9	(14)
	conflict	**26**	(2)	3	(3)	−2	(3)	6	(3)	−3	(3)	−4	(3)	1	(3)
switching	general RT	**1031**	(18)	−44	(24)	−**64**	(24)	−3	(24)	19	(24)	17	(23)	−16	(28)
	conflict	**130**	(8)	**17**	(10)	−**21**	(10)	1	(11)	2	(11)	11	(12)	−5	(11)

*Note*. *N* = 144, approx. *d*.*f*. = 137. WMC Working Memory Capacity, IQ General Intelligence, STS: SAS = Social Assessment, SRE = Self-Reinforcement, SCR = Self-Criticism, SMA Self-Management. Significant coefficients (*p* < 0.05) are printed bold.

As expected, coefficients for conflict in all conflict tasks were positive, ranging from 6 ms (Simon) to 98 ms (arrow flanker), indicating that responses are slower for incongruent compared to congruent trials. We also observed large switch costs (109 ms and 130 ms for the non-verbal and the verbal switching task, respectively). Most interesting were of course the cross-level interactions. We predicted a contribution of WMC to task performance in all tasks. When entered as a level-2 predictor together with general intelligence, however, WMC did not predict reduced general RT or RT difference scores in any of the four conflict tasks and both switching tasks. For participants scoring high in IQ we predicted better performance in our verbal tasks. When entered as a level-2 predictor together with WMC, general intelligence predicted reduced general RT for the Simon, arrow flanker, Stroop and both switching tasks, as well as reduced conflict for arrow flanker and Stroop task.

For participants habitually engaging in inner speech processes we predicted reduced conflict effects and switch costs. Of the VISQ subscales (Table 2), evaluative and motivational inner speech predicted the conflict parameter in the Simon task as well as the arrow flanker task in the expected direction. For participants with a subscale score one standard deviation above the mean the Simon effect was 6 ms smaller and the arrow flanker was  10 ms smaller. Of the STS subscales (Table 3), Self-criticism predicted the conflict parameter in the Simon task whereas Self-Management predicted the conflict in the arrow flanker task in the expected direction. For participants with a subscale score one standard deviation above the mean (compared to an average score), the Simon effect was 7 ms smaller and the arrow flanker was 18 ms smaller. There were no effects of the other subscales of VISQ or STS on the conflict contrast or the switch costs. Parameter estimates for the inner speech scales were comparable when the ADS was added as an additional predictor at level-2.

## Discussion

In our study, we were interested to what degree inner speech habits (i.e., self-instructions and evaluation) benefit cognitive control. To this end, we assessed performance in four commonly used conflict tasks using symbolic and verbal stimuli, as well as two task switching paradigms and inner speech habits using three questionnaires. Although we could replicate the factor structure within the respective questionnaires, we could not establish a common factor across questionnaires representing overall frequency of inner speech. This supports the view of content-specific inner speech serving separate functions, and we therefore examined the predictive value of individual subscales for our performance measures.

As for the questionnaires, we could also not establish a common cognitive control factor for the conflict tasks and the switching tasks. There was considerable variability in individual task performance across participants, and although congruency effects and switch costs varied in size and reliability across tasks, they were sizable and reliable enough for correlations to emerge, given the sample size. Yet, substantial positive correlations were found only for the arrow flanker and Stroop task and both switching tasks. The otherwise unrelated conflict parameters point to a diverse range of specific abilities necessary to solve response requirements in these tasks, making only two latent factors less likely to account for individual differences in cognitive control across our tasks^32^.

In consequence, we adopted piecewise analysis strategy and found that inner speech habits related to motivational and evaluative aspects of performance predict better performance in the Simon as well as the arrow flanker tasks, supporting our hypothesis that depending on context, task-related inner speech habits do have an implication over and above communication in that participants are less affected by response conflict and henceforth experience less interference and performance costs. Furthermore, we also predicted that those influences should be more visible in tasks employing non-verbal, symbolic stimuli such as shapes or arrows which do not foster verbal strategies because of stimulus material. This prediction was confirmed for two tasks (Simon and arrow flanker) in which we found influences of inner speech on. Unlike other studies that were mainly concerned with the both detrimental as well as supportive influence of instructed inner speech on concurrent actions^8,9,12,33^, our study discerns the benefits of inner speech habits as a language-related personality trait for conflict resolution.

Interestingly (and importantly), influences of motivational and evaluative inner speech persisted when we controlled for working memory capacity and intelligence, in line with our finding that inner speech habits were unrelated to and hence its benefits cannot be explained by greater working memory capacity or higher intelligence. We speculate that the questionnaire items assessing motivational and evaluative inner speech indeed capture more local processes of interference control or performance monitoring. These more local processes directly translate into actual performance, whereas global characteristics (i.e., processing speed as assessed as general RT level) are linked to overarching constructs such as intelligence and working memory capacity.

However, although interesting our results still suffer from limitations. Our first concern refers to the assessment of different contents of inner speech. In the questionnaires used, most of the items that offer some exemplification are rather broad and unspecific (i.e., “I talk through my plans”). The items within the evaluative and motivational inner speech subscale, however, are quite concrete and performance-related. This concreteness might have been necessary for the observed predictive value regarding task performance and may explain the absent effects for the other subscales. Second, out of the four conflict tasks and the switching tasks the influence of inner speech habits was restricted the Simon task as well as the arrow flanker task. Those tasks go along with strong response activation features for irrelevant responses in case of incongruent trials. Next, both tasks consist of only four stimulus-response (S–R) episodes (i.e., left and right pointing arrows as targets and flankers) that might allow for rapid automatization within a small number of trials. With a higher number of S–R epsiodes, recurrently retrieving the instructions and activating the phonological representation of the relevant stimulus features may render motivational inner speech and thereby individual differences less likely. Our third concern refers to the missing influence of working memory. To ensure instruction-compliant behavior, we displayed the relevant S–R mapping throughout the tasks, possibly reducing working memory load. This might have reduced variability among participants as working memory capacity was less relevant and therefore equalized it as predictor of performance.

To summarize, in our study being concerned with the influence of habitual use of inner speech on cognitive control, we found benefits of motivational and evaluative inner speech for performance in tasks allowing for fast automatization and focused attention such as the Simon task and the arrow flanker task, incremental to the predictive value of intelligence and working memory capacity. Future studies are necessary to examine whether more or less concrete contents of inner speech are helpful, over and above sequential self-instructions, and if the benefits extend to more complex tasks or tasks affording sustained attention.

## Methods

The study was carried out in accordance with guidelines formulated in the Declaration of Helsinki. The Ethics Committee of the German Society for Psychology approved the study (MG 042014). Informed consent was secured from all participants.

### Participants

502 participants were sampled from the participants’ pool of the Julius-Maximilians University of Würzburg. 32 participants had to be excluded due to suspiciously fast responding, false responses to two careless-responder catch items or because they opted for “do not use my data”. Data of the remaining sample (N = 470, 150 female, mean age = 24.6 years, *SD* = 4.73 years) were used to calculate the psychometric properties and the confirmatory factor analyses of the questionnaires. Of those 470 participants, 163 took part in the lab study and received course credit or €25. Questionnaire data of these participants were comparable to the full sample (all $p < 0.01$ for equivalence tests^34^ with $d=0.5$). In eight participants, one of the ten experimental tasks crashed, yielding complete experimental data for 155 participants (38 male), with a mean age 24.5 years (SD 3.6 years). Due to experimenter error, IQ data were missing for 11 participants, reducing the sample size to 144 participants (36 male, mean age 24.5 years, SD 3.6 years) for analyses controlling for intelligence. All participants reported normal or corrected-to-normal vision.

### Questionnaires

After signing up for the study, participants were invited to fill in the following questionnaires: For inner speech habits we assessed the Varieties of Inner Speech Questionnaire^15^ (VISQ, 18 items); the Self Talk Scale^16^ (STS, 16 items), and the Inner Speech Scale^23^ (ISS, 18 items). The VISQ assesses inner speech habits along four dimensions (dialogic inner speech, condensed inner speech, other people in inner speech, and evaluative and motivational inner speech), whereas the STS and ISS are instruments coming from health psychology backgrounds and are used commonly to assess inner speech habits more in style of coping behavior. Additionally, we assessed short forms of the Big Five Inventory^35^ (BFI-S, 15 items), the General Depression Scale^36^ (“Allgemeine Depressions Skala – Kurzform”, ADS-K, 15 items) and the Edinburgh Handedness Inventory^37,38^ (EHI-SH, four items). The assessment was performed online using SosciSurvey^39^ and took about 15 to 20 min.

Reliability analyses of the inner speech questionnaire data were based on McDonalds *ω*^40^ and were performed using the R package “mbess^41^”. The reliability values of the VISQ subscales were acceptable to good, considering the length of the scales (four to five items each): For the subscale dialogic inner speech, *ω* = 0.72 [CI 0.68–0.76], for the subscale condensed inner speech, *ω* = 0.79 [CI 0.76–0.82], for the subscale addressing the other people in inner speech, *ω* = 0.86 [CI 0.83–0.88] and for the evaluative and motivational inner speech subscale, *ω* = 0.71 [CI 0.65–0.75]. The reliability values of the STS subscales were acceptable to good, Social Assessment: *ω* = 0.81 [CI 0.78–0.84], Self-Reinforcement: *ω* = 0.82 [CI 0.79–0.85], Self-Criticism: *ω* = 0.76 [CI 0.72–0.79], Self-Management: *ω* = 0.75 [CI 0.70–0.78].

### Measures of IQ and Working Memory Capacity

The short version of the Berliner Intelligenz Struktur Test^42^ (BIS) comprises 16 paper-pencil tests that can be aggregated to a measure of general intelligence (AI for “Allgemeine Intelligenz”). Raw scores were transformed to Standard scores using the norms given in the manual. The Working Memory Capacity Battery^43^ (WMC) consists of four tasks, an updating task (memory updating, MU), two span tasks (OS; sentence span, SS), and a spatial task (spatial short-term memory, SSTM). Due to a coding error in the two span tasks only the maintenance scores were saved but no data for the processing component (i.e., judging an equation as correct or incorrect). Mean percent correct values were computed for all four tasks. Since the scores were found to load on a single latent variable, we ran a principal component analysis of the mean percent correct values to obtain a measure of the shared variance of the tasks. The first two eigenvalues were 2.54 and 0.81; accounting for 63.4% and 20.2% of the variance. Thus, one factor was retained and its standardized factor score was used for the analyses.

### Tasks

Tasks were programmed and run using Eprime 2.0.10.356 Professional (Psychology Software Tools, Pittsburgh, PA). Stimulus color (unless indicated otherwise) was white on darkgrey background. All tasks started with an instruction that comprised a brief description of task affordances followed by practice trials comprising all stimuli to be encountered in the task and their assigned responses. In case of open questions, the experimenter explained the task orally. All tasks and blocks within a task were started self-determined. The new tasks were announced before the instruction sheets were displayed. All tasks consisted of 32 practice trials and 192 test trials (three blocks of 64 trials for the switching tasks and two blocks of 96 test trials for all other tasks). All trials started with a fixation cross (Calibri 44 points) or in case of task switching with the cue for 500 ms and were followed by a feedback using a smiling or frowning face (2.54 cm in diameter) dependent on response accuracy for 250 ms followed by a blank for 500 ms. S–R mappings for the actual task at hand were displayed at the bottom of the screen. Different response keys were used for each task and no upper response time limit was given. After each block, participants were informed about their percentage of errors and invited to take a short rest. All participants performed the following tasks in two blocks of three tasks each: The non-verbal tasks block consisted of the Arrow Flanker, Simon and pictorial Task Switching. The verbal tasks block consisted of the Letter Flanker, Stroop and verbal Task Switching.

#### Non-verbal tasks block

In the arrow flanker task participants had to indicate the direction (left vs. right) of a central arrow that was flanked by two additional arrows to the right and the left (i.e., distractors). These distractors could either point in the same direction as the central arrow (i.e., congruent trials) or in the opposite direction (i.e., incongruent trials). The imperative stimulus consisting of five arrows (“>” or “<” in Calibri 44 points), presented centrally until response with keys “X” and “M” of a standard German QWERTZ-keyboard was given.

For the Simon task, participants had to indicate whether a given stimulus was a square or a diamond using the “A” or the “L” key. The imperative stimulus was presented 7 cm to the left or the right of the fixation cross. For the Simon task, congruent trials (i.e., location of the imperative stimulus and required response matched) were equally frequent as incongruent trials (i.e., location of the imperative stimulus and required response did not match). The imperative stimulus, a diamond or a square with 2.54 cm × 2.54 cm edge length was presented either left or right of the fixation cross and remained visible until response.

In the pictorial task switching task, participants had to classify shapes either as round or angular or whether they were symmetrical or not. Six shapes were chosen for this task and comprised an ellipse, a cloud, a lake-like shape, a drop as well as a square, a hexagon, a freely designed shape without meaning but no curves and an asymmetric star. All shapes covered an area of 4 × 5 cm on the screen and were presented centrally. The cues (3 × 3 cm) indicating which task had to be performed were a circle superimposed on a triangle for the round/angular task and a heart with vertical straight line for the symmetry task. Assigned response keys were “1” in case of a symmetrical or angular object and “9” for asymmetrical and round objects. A trial started with the presentation of the cue at the top for 500 ms followed the imperative stimulus. The cue, S–R mapping and stimulus remained visible until the response. Task switches, i.e. trials in which the current task was different from the task in the previous trial were equally frequent as task repetitions in which the task stayed the same across two consecutive trials.

#### Verbal tasks block

In the Stroop task participants were asked to indicate the color in which as color word was written. In incongruent trials, the print color did not match the meaning of the color word (i.e., the word “red” printed in blue), for congruent trials print color and word meaning matched (i.e,, “red” printed in red). Response keys were “Y”, “V”, “N” and “−” for the colors red, blue, green and yellow, respectively. Stimuli were colored color words (“rot”, “blau”, “grün” and “gelb” in German for red, blue, green and yellow) written in Calibri 44 points and presented centrally. Each participant encountered four congruent and four incongruent stimuli but all incongruent stimuli were presented counterbalanced over participants. Half of the trials participants encountered were congruent and the other half incongruent.

In the letter flanker task, participants were required to classify the central letter out of a string comprised of five letters as consonant or vowel. The two letters to the left or right of the central letter belonged either to the same category (i.e., “A” being flanked by “E”, congruent) or to the other category (i.e., “E” being flanked by “F”, incongruent). Participants responded to vowels by using the “K” key of the keyboard and to consonants by using the “D” key of the keyboard. Congruent and incongruent trials were equally frequent.

In the verbal task switching task participants were asked to judge a noun whether it was concrete or abstract or whether it went along with a female (f) or male (m) article in German. Stimuli words were love (f), fidelity (f), cloud (f), flower (f), pride (m), frustration (m), tree (m), and pot (m). All words were written in Calibri 44 points and presented centrally. In German, all words consisted of 4–5 letters. Cues were the words “Artikel” (article) for the male/female article categorization and “Nomen” (noun) for the concrete vs. abstract categorization. Participants used the keys “Q” for male and abstract nouns and the “P” key for female and concrete nouns. Procedure was similar to the non-verbal task switching task.

#### Reliability of task parameters

To estimate split-half reliability of the task parameters, multilevel models (cf. Results section) were run independently for each task with separate general RT and conflict parameters for each half of the respective task. Within all tasks, split-half reliabilities of the general RT parameters were excellent (all $r > 0.9$). Split-half correlations of the conflict parameters were more varied. Reliabilities were acceptable to excellent for the nonverbal tasks (arrow flanker $r=0.74$, Simon $r=0.87$, pictorial switch $r=0.90$). Of the verbal tasks, the Stroop and switching task had barely acceptable reliabilities ($r=0.73$ and $r=0.64$, respectively), wheres for the letter flanker task both halfs were essentially uncorrelated ($r=-\,0.09$).

### Procedure

Participants were tested in pairs on two desktop PCs, running Windows 7 connected to 19” flat screen monitors with a 1280 × 1024 pixel resolution and USB connected standard German QWERTZ keyboards. Order of tasks blocks and WMC battery was counterbalanced across participants, resulting in about 40 participants for the four conditions (verbal vs. nonverbal tasks block first; WMC battery before or after the tasks). The three Stroop mappings were evenly distributed across the four conditions. Overall, testing amounted to 3 hours.

#### Data Trimming

We removed all practice blocks as well as errors (4.0%) and trials following an error (3.9%). Furthermore, the first trial in every block was also removed as were all trials with reaction times shorter than or equal to 50 ms and trials with reaction times longer than 2500 ms (1.3%). The trimming procedure was identical across all analyses.

## Supplementary information

Supplementary information

## Data Availability

The datasets generated during and/or analysed during the current study are available in the OSF repository, https://osf.io/9kx7y/?view_only=5c7a84693b384d4a87b71950338d9dd0.
