# Supplementary material for: Talking matters – evaluative and motivational inner speech use predicts performance in conflict tasks
Source: Sci Rep. 2019 Jul 2;9:9531. doi: 10.1038/s41598-019-45836-2 (PMC6606602; doi:10.1038/s41598-019-45836-2)
Supplement: Supplementary file 1 — Supplementary information [file 41598_2019_45836_MOESM1_ESM.pdf]

Talking matters – evaluative and motivational inner speech use predicts performance in conflict tasks: Supplementary information

Miriam Gade[1,3,*] **and Marko Paelecke**[2]

[1]Catholic University of Eichstätt-Ingolstadt, Department of Psychology, Eichstätt, D–85071, Germany
[2]Julius Maximilians University Würzburg, Department of Psychology I, Würzburg, D–97070, Germany
[3]Medical School Berlin, Department of Sciences, Berlin, D-12247, Germany
[*]miriam.gade@medicalschool-berlin.de

Supplementary Results

Correlations between predictors, control variables and task parameters

Pearson correlations between predictors, control variables and individual participants' task parameters are depicted in Tables S1–S4. Task parameters (for general RT and error probability as well as conflict) are empirical Bayes estimates of the randomly varying level-2 coefficients from a multilevel model that included only Level 1 predictors (cf. Results section), i.e., a dummy variable for each task coding general RT and a task-specific contrast that modeled the RT difference between congruent and incongruent trials with in each task.

Predicting task performance: Error rates

To analyze the effect of inner speech habits on error probabilities on the trial level, we used Hierarchical Generalized Linear Modeling (HGLM) with HLM7. Level 1 predicted the probability of an error (coded as 1) compared to a correct response (coded as 0) using the logit link function from task-specific dummy variables coding general error probability and task-specific contrasts that modeled the difference between congruent and incongruent trials with in each task. Level 2 predicted between-subject variability of the task-specific parameters coding general RT and conflict from the particiants WMC, IQ as well as their inner speech questionnaire scores. Scores of all level-2 predictors were entered as standard scores ($M = 0$, $SD = 1$). We ran two separate analyses for the subscale scores of the VISQ and STS. The coefficients for fixed effects of general error probability and conflict for each task are depicted in Tables S5 and S6.

In both subject-specific models, the general odds of an error in all tasks were very low (all intercepts $\beta_{00} < -3$, all odds ratios $OR < 0.05$). The coefficients for the congruency contrasts were positive for all tasks and significant except for the Stroop task, indicating an higher odds ratio for incongruent compared to congruent trials. WMC did predict a reduced general error probability in four of the six tasks, whereas IQ predicted an increased general error probability in three of the six tasks, pointing to a speed-accuracy trade-off for IQ. There were no significant effects of the VISQ subscale evaluative and motivational inner speech on the conflict parameter in the Simon task as well as the nonverbal flanker task. The sign of the cross-level interaction parameters, however, were in the same expected direction to those of the HLM analysis of response times, ruling out a speed-accuracy trade-off for this VISQ subscale. There were no effects of the STS subscales on the conflict contrast.

Supplementary Table S1. Pearson correlations between questionnaire scores in the online sample.

Measure	Scale	1	2	3	4	5	6	7	8	9	10	11	12	13	14
VISQ															
	1. DLG	—													
	2. CND	.25	—												
	3. OTP	.27	.20	—											
	4. E/M	.44	.28	.25	—										
STS															
	5. SAS	.29	.08	.21	.21	—									
	6. SRE	.30	.10	.14	.24	.54	—								
	7. SCR	.33	.12	.15	.41	.50	.48	—							
	8. SMA	.31	.05	.16	.32	.65	.51	.50	—						
BFI–S															
	9. N	.07	.07	.17	.17	.12	.08	.21	.16	—					
	10. E	–.05	.03	.01	–.05	–.18	–.04	–.13	–.18	–.22	—				
	11. O	.12	.04	.15	.09	–.01	.02	–.03	–.01	.03	.15	—			
	12. A	–.06	.00	–.02	–.05	–.02	.00	–.07	–.10	–.15	.04	.04	—		
	13. C	–.09	–.05	.02	–.04	–.01	.01	–.03	–.01	–.07	.06	.07	.20	—	
ADS–K															
	14. D	.17	.07	.22	.18	.19	.07	.20	.17	.44	–.22	.07	–.11	–.22	—

Note. $N = 470$. VISQ: DLG = Dialogic inner speech, CND = Condensed inner speech, OTP = Other people in inner speech, E/M = Evaluative and motivational inner speech. STS: SAS = Social Assessment, SRE = Self–Reinforcement, SCR = Self–Criticism, SMA Self–Management. BFI–S: N = Neuroticism, E = Extraversion, O = Openness A = Agreeableness, C = Conscientiousness. ADS–K: D = Depression. Critical values of $|r|$ for $p < .05$ are $r_c = 0.08$ (one–tailed) and $r_c = 0.09$ (two–tailed).

Supplementary Table S2. Pearson correlations between predictors and control variables in the lab sample.

Measure	Scale	1	2	3	4	5	6	7	8	9	10	11	12	13	14	15	16
VISQ																	
	1. DLG	—															
	2. CND	.21	—														
	3. OTP	.16	.17	—													
	4. E/M	.40	.29	.21	—												
STS																	
	5. SAS	.17	.05	.16	.02	—											
	6. SRE	.32	.15	.10	.14	.52	—										
	7. SCR	.25	.21	.18	.34	.42	.50	—									
	8. SMA	.26	.15	.17	.23	.62	.53	.51	—								
BFI-S																	
	9. N	–.18	.07	.04	.05	–.14	–.08	.00	–.05	—							
	10. E	.03	.06	.08	.09	–.17	–.01	–.10	–.17	–.16	—						
	11. O	.11	.09	.12	.16	–.08	.01	.01	–.15	.02	.12	—					
	12. A	.00	–.08	–.01	.03	.01	–.03	–.08	.02	–.12	.11	–.03	—				
	13. C	–.12	–.09	.09	–.05	–.01	.14	.09	.02	–.12	.03	–.06	.24	—			
ADS-K																	
	14. D	.10	.00	.15	.20	.05	.00	.16	.12	.36	–.18	.11	–.19	–.16	—		
WMC Battery																	
	15. WMC	.06	.00	–.12	.08	–.09	.01	.07	–.03	.00	–.02	–.09	.05	–.01	–.03	—	
BIS																	
	16. IQ	.15	.06	.00	.18	–.13	–.07	.03	–.14	–.08	.04	–.06	–.01	.05	–.08	.50	—

Note. $N = 144$. VISQ: DLG = dialogic inner speech, CND = condensed inner speech, OTP = other people in inner speech, E/M = evaluative and motivational inner speech. STS: SAS = Social Assessment, SRE = Self-Reinforcement, SCR = Self-Criticism, SMA Self-Management. WMC Working Memory Capacity, BIS: IQ = General Intelligence. Critical values of $|r|$ for $p < .05$ are $r_c = 0.14$ (one-tailed) and $r_c = 0.16$ (two-tailed).

Supplementary Table S3. Pearson correlations of individual participants' task parameters for general response time (RT) and conflict with participants WMC, IQ as well as their VISQ and STS subscale scores.

task					VISQ				STS			
		WMC	IQ	DLG	CND	OTP	E/M	SAS	SRE	SCR	SMA
nonverbal											
Simon	general RT	–.27	–.34	.00	.14	.12	.02	.07	.11	.13	.12
	conflict	.07	–.05	–.06	–.05	.05	–.16	–.01	–.05	–.11	–.06
flanker	general RT	–.24	–.38	.01	.20	.17	–.09	.11	–.01	–.05	–.02
	conflict	–.27	–.34	.01	.16	.16	–.15	.13	–.06	–.12	–.09
switching	general RT	–.16	–.25	.05	.17	.09	.07	.06	.09	.08	.07
	conflict	.08	–.04	.14	.18	.07	.10	–.07	.03	.07	.00
verbal											
Stroop	general RT	–.24	–.39	–.05	.08	.13	–.05	–.01	.09	.00	.04
	conflict	–.29	–.41	–.03	.14	.16	–.06	.06	.07	.03	.04
flanker	general RT	–.29	–.27	.00	.04	.07	–.03	.13	.14	.08	.15
	conflict	–.12	–.14	.05	.08	.08	–.02	.10	–.06	–.14	–.06
switching	general RT	–.30	–.35	.07	.16	.16	.07	.07	.09	.05	.05
	conflict	.04	–.15	.18	.21	.21	.12	.03	.07	.10	.04

Note. $N = 144$. WMC Working Memory Capacity, IQ General Intelligence. VISQ: DLG = dialogic inner speech, CND = condensed inner speech, OTP = other people in inner speech, E/M = evaluative and motivational inner speech. STS: SAS = Social Assessment, SRE = Self-Reinforcement, SCR = Self-Criticism, SMA Self-Management. Task parameters are empirical Bayes estimates. Critical values of $|r|$ for $p < .05$ are $r_c = 0.14$ (one-tailed) and $r_c = 0.16$ (two-tailed).

Supplementary Table S4. Pearson correlations of individual participants' task parameters for general error probability (EP) and conflict with participants WMC, IQ as well as their VISQ and STS subscale scores.

task				VISQ				STS			
		WMC	IQ	DLG	CND	OTP	E/M	SAS	SRE	SCR	SMA
nonverbal											
Simon	general EP	–.13	.08	–.01	–.14	.00	–.09	–.11	–.17	–.18	–.16
	conflict	.00	–.17	–.02	–.04	–.07	–.14	.14	.12	.00	.09
flanker	general EP	–.13	.14	–.01	–.10	.07	–.05	–.16	–.24	–.18	–.22
	conflict	.06	–.08	–.03	–.10	–.17	–.17	.11	.08	–.04	.10
switching	general EP	–.16	–.01	–.04	–.21	–.08	–.14	–.11	–.08	–.15	–.16
	conflict	.15	.01	.00	.11	.10	.13	.01	–.04	–.01	.01
verbal											
Stroop	general EP	–.07	.08	–.01	–.12	.04	–.10	–.15	–.14	–.16	–.21
	conflict	.01	–.09	.02	.02	–.04	–.04	.11	.08	.09	.09
flanker	general EP	–.13	.02	–.03	–.12	–.01	–.14	–.09	–.09	–.10	–.15
	conflict	.01	.16	.06	.12	.09	.05	–.01	–.13	–.02	–.03
switching	general EP	–.14	.08	–.06	–.12	–.02	–.10	–.14	–.13	–.14	–.20
	conflict	.10	–.01	–.07	–.12	–.03	.07	–.11	–.08	–.08	–.08

Note. $N = 144$. WMC Working Memory Capacity, IQ General Intelligence. VISQ: DLG = dialogic inner speech, CND = condensed inner speech, OTP = other people in inner speech, E/M = evaluative and motivational inner speech. STS: SAS = Social Assessment, SRE = Self-Reinforcement, SCR = Self-Criticism, SMA Self-Management. Task parameters are empirical Bayes estimates. Critical values of $|r|$ for $p < .05$ are $r_c = 0.14$ (one-tailed) and $r_c = 0.16$ (two-tailed).

Supplementary Table S5. Coefficients (robust standard errors) for fixed effects of general error probabilities (EP) and conflict for each task, with participants WMC, IQ as well as their VISQ subscale scores as simultaneous predictors in the Level-2 model.

task									VISQ							
			Intercept		WMC		IQ		DLG		CND		OTP		E/M	
nonverbal																
	Simon	general EP	**–3.62**	(0.08)	**–0.21**	(0.08)	**0.20**	(0.10)	0.05	(0.09)	–0.11	(0.10)	–0.01	(0.09)	–0.03	(0.09)
		conflict	**0.41**	(0.09)	0.17	(0.12)	**–0.34**	(0.13)	0.04	(0.09)	0.04	(0.11)	0.01	(0.09)	–0.15	(0.11)
	flanker	general EP	**–4.44**	(0.10)	**–0.27**	(0.11)	**0.25**	(0.12)	–0.03	(0.10)	–0.05	(0.10)	0.17	(0.09)	–0.03	(0.12)
		conflict	**2.26**	(0.15)	0.05	(0.12)	0.14	(0.13)	0.07	(0.15)	–0.18	(0.14)	–0.25	(0.13)	–0.22	(0.17)
	switching	general EP	**–3.11**	(0.08)	**–0.21**	(0.09)	0.10	(0.09)	0.05	(0.09)	**–0.20**	(0.09)	–0.08	(0.08)	–0.10	(0.10)
		conflict	**0.69**	(0.06)	0.05	(0.05)	–0.05	(0.06)	0.05	(0.06)	–0.08	(0.06)	0.11	(0.06)	0.03	(0.07)
verbal																
	Stroop	general EP	**–4.03**	(0.08)	–0.03	(0.09)	0.09	(0.11)	0.02	(0.10)	–0.11	(0.08)	0.09	(0.10)	–0.12	(0.10)
		conflict	0.13	(0.08)	0.14	(0.09)	0.07	(0.09)	–0.02	(0.09)	–0.09	(0.08)	–0.04	(0.08)	–0.07	(0.10)
	flanker	general EP	**–3.60**	(0.08)	–0.13	(0.11)	0.07	(0.10)	0.07	(0.08)	–0.06	(0.08)	0.04	(0.08)	–0.15	(0.09)
		conflict	**0.86**	(0.08)	–0.11	(0.08)	0.11	(0.11)	0.03	(0.08)	0.11	(0.09)	–0.03	(0.09)	0.02	(0.09)
	switching	general EP	**–3.24**	(0.08)	**–0.26**	(0.10)	**0.26**	(0.10)	–0.06	(0.08)	–0.08	(0.08)	–0.03	(0.09)	–0.05	(0.09)
		conflict	**0.57**	(0.07)	**0.07**	(0.08)	–0.05	(0.08)	–0.10	(0.07)	–0.08	(0.07)	–0.04	(0.08)	**0.16**	(0.08)

Note. $N = 144$, approx. $d.f. = 137$. WMC Working Memory Capacity, IQ General Intelligence, VISQ: DLG = dialogic inner speech, CND = condensed inner speech, OTP = other people in inner speech, E/M = evaluative and motivational inner speech. Significant coefficients ($p < .05$) are printed bold.

Supplementary Table S6. Coefficients (robust standard errors) for fixed effects of general error probabilities (EP) and conflict for each task, with participants WMC, IQ as well as their STS subscale scores as simultaneous predictors in the Level-2 model.

task									STS							
			Intercept		WMC		IQ		SAS		SRE		SCR		SMA	
nonverbal																
	Simon	general EP	**–3.63**	(0.08)	**–0.18**	(0.09)	0.19	(0.10)	0.01	(0.11)	–0.11	(0.11)	–0.13	(0.10)	0.05	(0.12)
		conflict	**0.39**	(0.09)	0.19	(0.12)	**–0.33**	(0.13)	0.21	(0.13)	0.05	(0.12)	–0.15	(0.13)	0.00	(0.14)
	flanker	general EP	**–4.44**	(0.10)	**–0.30**	(0.11)	0.22	(0.11)	–0.03	(0.13)	–0.26	(0.14)	0.01	(0.12)	–0.09	(0.14)
		conflict	**2.24**	(0.15)	0.15	(0.13)	0.07	(0.15)	0.11	(0.22)	0.11	(0.21)	–0.32	(0.18)	0.07	(0.22)
	switching	general EP	**–3.11**	(0.08)	**–0.19**	(0.09)	0.06	(0.09)	–0.04	(0.11)	0.07	(0.10)	–0.11	(0.11)	–0.11	(0.13)
		conflict	**0.69**	(0.06)	0.05	(0.06)	–0.03	(0.06)	0.11	(0.09)	0.04	(0.07)	–0.11	(0.08)	–0.11	(0.10)
verbal																
	Stroop	general EP	**–4.03**	(0.08)	–0.04	(0.09)	0.06	(0.11)	–0.06	(0.12)	0.04	(0.11)	–0.06	(0.12)	–0.18	(0.13)
		conflict	0.13	(0.08)	0.14	(0.09)	0.05	(0.09)	–0.12	(0.10)	–0.05	(0.12)	0.05	(0.11)	0.07	(0.12)
	flanker	general EP	**–3.60**	(0.08)	–0.13	(0.10)	0.04	(0.10)	0.02	(0.10)	0.05	(0.09)	0.04	(0.09)	–0.13	(0.13)
		conflict	**0.87**	(0.08)	–0.08	(0.09)	0.11	(0.11)	0.16	(0.13)	–0.18	(0.12)	0.01	(0.12)	–0.01	(0.13)
	switching	general EP	**–3.24**	(0.08)	**–0.25**	(0.10)	0.21	(0.11)	–0.06	(0.10)	0.02	(0.11)	–0.04	(0.12)	–0.15	(0.12)
		conflict	**0.57**	(0.07)	0.09	(0.08)	–0.06	(0.09)	–0.05	(0.10)	–0.07	(0.10)	–0.02	(0.10)	–0.01	(0.10)

Note. $N = 144$, approx. $d.f. = 137$. WMC Working Memory Capacity, IQ General Intelligence, STS: SAS = Social Assessment, SRE = Self-Reinforcement, SCR = Self-Criticism, SMA Self-Management. Significant coefficients ($p < .05$) are printed bold.
